# Review of Current Systemic Therapy and Novel Systemic Therapy for Pancreatic Ductal Adenocarcinoma

**DOI:** 10.3390/curroncol30060404

**Published:** 2023-05-26

**Authors:** Humaira Sarfraz, Aditi Saha, Khushali Jhaveri, Dae Won Kim

**Affiliations:** Department of Gastrointestinal Oncology, Moffitt Cancer Center, Tampa, FL 33612, USA; humaira.sarfraz@moffitt.org (H.S.); aditi.saha@moffitt.org (A.S.); khushali.jhaveri@moffitt.org (K.J.)

**Keywords:** pancreatic adenocarcinoma, chemotherapy

## Abstract

Background: This review aims to describe the systemic treatment options for pancreatic ductal adenocarcinoma and includes a summary of the current treatments as well as the ongoing clinical trials which may be efficacious in the treatment of this aggressive malignancy. Methods: A literature review was performed using MEDLINE/PubMed between August 1996 and February 2023. The reviewed studies are categorized into these categories: current standard of care treatments, targeted therapies, immunotherapy and clinical trials. The current treatment modality for the treatment of advanced pancreatic cancer is mainly systemic chemotherapy. Results: The introduction of polychemotherapy regimens including gemcitabine/nab-paclitaxel and FOLFIRINOX (oxaliplatin, irinotecan, folinic acid and fluorouracil) has improved the clinical outcome of advanced pancreatic cancer. For further improvement in clinical outcomes, several novel approaches have been extensively studied in pancreatic cancer. The review discusses the current standard chemotherapy regimen and the novel treatment options in the field. Conclusions: While there are novel treatments being explored for metastatic pancreatic, it remains a debilitating and aggressive disease with high mortality that warrants continued efforts to advance therapeutic options.

## 1. Introduction

The projected total number of cases of pancreatic cancer diagnosed in the United States for 2023 are 64,050, with the estimated deaths being 50,550 for the calendar year [1]. Meanwhile, every year, the incidence of pancreatic cancer is slowly going up at a rate of 0.5–1% and it is speculated that, by 2030, pancreatic cancer will be the second most common cause of death [2]. The sole potentially curative method is regarded as surgical removal. However, only a limited number of patients are able to seek surgical resection with the rest detected with advanced unresectable disease or metastatic disease. Pancreatic cancer has an aggressive clinical course and a poor prognosis. Moreover, it continues to be a therapeutic challenge with limited options, limited efficacy and limited tolerance to treatment. The introduction of multiagent chemotherapy regimens have remarkably improved the clinical outcome of advanced pancreatic cancer. However, five-year survival rates for metastatic pancreatic cancer are less than 5%. To improve the clinical outcome of pancreatic cancer, a better understanding of the benefits and limitations of systemic treatment options is needed. This review summarizes the current standard systemic approaches and novel systemic approaches to improve our understanding of pancreatic cancer management.

## 2. Materials and Methods

A literature review was performed using MEDLINE/PubMed between August 1996 and February 2023. A total of 71 articles are included in this review. The reviewed studies are categorized into these categories: current standard of care treatments (37 articles), targeted therapies (4 articles), immunotherapy (7 articles) and clinical trials (23 articles).

## 3. Results

### 3.1. Resectable and Borderline Resectable Disease

The primary intent of neoadjuvant treatment in pancreatic adenocarcinoma is to eliminate possible micrometastasis and maximize the chances for margin-negative (R0) surgery. For patients with borderline resectable pancreatic cancer (BRPC), many studies have shown the clinical advantage associated with neoadjuvant therapy. A total of 1980 patients with BRPC were evaluated in a large retrospective study and received neoadjuvant therapy. These patients had a significantly higher likelihood of node-negative (59% versus 24%, *p* < 0.001) and surgery with negative margins (82% versus 56%, *p* < 0.001). In comparison to patients who received adjuvant monotherapy, the patients who received neoadjuvant therapy were noted to have a better median overall survival of 24.7 months versus 19.6 months (*p* < 0.0001) [3]. However, one limitation of the study was that, since the information was derived from the National Cancer Database, details on the specific type and duration of chemotherapy were not available.

In a single arm phase 2 study of BRPC, 48 patients received eight cycles of FOLFIRINOX followed by chemoradiation. R0 resection was achieved in 65% of the 48 eligible patients and in 97% of the 32 patients who had surgical resection [4]. Although the definition of R0 resection remains debatable, the R0 resection rates of this study were higher than the historically reported rates of 41–72% [5].

The ESPAC5 study was a multicenter, phase 2 study which attempted to evaluate the feasibility and effectiveness of neoadjuvant therapy and immediate surgery. The chemotherapy combinations used included gemcitabine plus capecitabine, FOLFIRINOX (oxaliplatin, irinotecan, folinic acid and fluorouracil) or capecitabine-based chemoradiation [6]. Amongst the patients who had upfront surgery, 21 patients (68%) underwent surgical resection, compared to the 7 patients (23%) in the neoadjuvant therapy group who had surgical resection (*p* = 0.33). The rate of R0 resection were greater in the neoadjuvant arm compared to upfront surgery; 23% vs. 14%, respectively (*p* = 0.49). Furthermore, the neoadjuvant approach was associated with improved overall survival. The study demonstrated one year survival rates of 39% in the upfront surgery arm, 60% for the capecitabine-containing chemoradiotherapy group, 78% in the gemcitabine plus capecitabine arm and 84% for FOLFIRINOX (*p* = 0.0028). Amongst the 28 patients who underwent upfront surgery, 7% were noted to have grade 3 or greater toxicity events, while 19 patients (34%) in the neoadjuvant therapy arm experienced these events. The most commonly noted toxicity events included neutropenia, infection and hyperglycemia. Thus, the study shows that the supplementation of neoadjuvant therapy in patients with borderline resectable pancreatic ductal carcinoma improves the clinical outcomes.

While neoadjuvant chemotherapy in patients with BRPC, has been shown to have many benefits, the efficacy of neoadjuvant therapy in resectable pancreatic cancer is still to be determined. A randomized phase 2 clinical trial assessed the role of neoadjuvant chemotherapy with modified FOLFIRINOX and gemcitabine/nab-paclitaxel in resectable pancreatic cancer (PC) [7]. Initial chemotherapy was not found to prolong OS significantly when compared to results from previous studies with adjuvant chemotherapy. Furthermore, in patients with resectable PC in this study, both FOLFIRINOX and gemcitabine/nab-paclitaxel showed comparable outcomes.

Currently, a phase 3 study of neoadjuvant FOLFIRINOX versus adjuvant FOLFIRINOX in resectable PC (NCT04340141) is ongoing in order to define the role of upfront chemotherapy in resectable PC. The major phase 2/3 clinical trials for resectable/borderline resectable disease are summarized in Table 1.

### 3.2. Locally Advanced Disease

Cases with locally advanced pancreatic cancer (LAPC) comprise 30% of the initial diagnostic presentations for pancreatic cancer. By virtue of the extensive vascular involvement, LAPC is considered surgically unresectable. Although a multidisciplinary approach is needed for locally advanced disease, the treatment of LAPC is quite challenging because patients frequently experience significant symptoms from local tumor burden, only 20% of patients become eligible for surgical resection after neoadjuvant therapy [11], and a majority of patients develop metastatic disease within one year [12]. Similarly, with BRPC, the efficacy of neoadjuvant chemotherapy has been evaluated for LAPC in multiple prospective studies.

The LAP07 study was a phase 3 randomized study which evaluated patients on induction chemotherapy with gemcitabine or gemcitabine in combination with erlotinib. This was followed by a subsequent randomization for patients with no evidence of disease progression after four months of treatment to continue with the same chemotherapy versus chemoradiotherapy [8]. Among patients receiving chemotherapy or chemoradiation as part of the second randomization, the median overall survival was similar (16.5 versus 15.2 months, *p* = 0.83). The median overall survival was 13.6 months in the gemcitabine arm versus 11.9 months in the gemcitabine plus erlotinib group (*p* = 0.09); hence, no statistical difference was found in the two arms. The patients who received chemoradiotherapy had a lower rate of local progression (32% versus 46%, *p* = 0.03). Curative intent surgery was performed in 18 patients (4%) in the study who had a median OS of 30.9 months. Amongst the patients undergoing surgery, 11 underwent R0 resection, 2 underwent R1 resection and, in 5 cases, the margin status remained unknown. One of the limitations of the study was that it was performed prior to the emergence of FOLFIRINOX and nab-paclitaxel as first line treatments; hence, compared to the current standard of care, a nonoptimal regimen was used in the study.

A multicenter phase 2 trial studied patients with locally advanced pancreatic cancer with no prior treatment who received the combination of nab-paclitaxel and gemcitabine [9]. The study’s main goal was to assess the median time to treatment failure, 9 months (90% CI 7.3–10.1), while the median PFS was 10.9 months and OS was 18.8 months. The best response was recorded as partial response in 36 patients (34%) while disease control was observed in 83 patients (78%). After six cycles of induction treatment, 17 (17%) underwent surgery with 7 R0 resection (7%). Notably, this was a single arm multicenter study without a central review of the imaging which might complicate the interpretation of the findings.

Chemotherapy with FOLFIRINOX and gemcitabine/nab-paclitaxel was compared in 126 randomized patients with LAPC. Patients on the arm receiving gemcitabine/nab-paclitaxel had superior 1-year OS (82.5% versus 77.4%). However, patients on the FOLFIRINOX arm had more improved 2-year OS (48.2% versus 39.7%) [10]. However, statistical significance was not observed here. Additional research is necessary to validate these findings.

The major phase II/III clinical trials for locally advanced disease are summarized in Table 1.

### 3.3. Resected Disease (Adjuvant Setting)

Despite theresection with curative intent in patients with pancreatic adenocarcinoma, clinical outcomes remain dismal with most of the patients having a relapse within 2 years of surgery [13]. Various chemotherapy regimens have been explored to reduce the risk of relapse and improve overall survival. Adjuvant systemic therapy options are standard of care for patients who have adequately recovered from surgery. The choice of treatment depends upon the response to neoadjuvant therapy, choice of neoadjuvant therapy, margins of resection, comorbidities and tolerance to treatment.

#### 3.3.1. Gemcitabine

The first multicenter, phase-randomized trial to explore the role of systemic therapy postoperatively was CONKO-001. Following complete tumor resection, the patients were randomized into 6 months of gemcitabine or observation. In the patients who received gemcitabine, the disease-free survival (DFS) was significantly longer with median DFS of 13.4 months versus 6.7 months in the observation arm (HR: 0.55; 95% CI: 0.44–0.69, *p*  <  0.001). The 10-year survival rate was 12.2% compared to 7.7%, respectively (HR: 0.76; 95% CI: 0.61–0.95, *p*  = 0 .01). Much higher relapses were noted within the first months following surgery in patients in the observation arm. Hence, the results favor the use of gemcitabine in this situation [14].

#### 3.3.2. Gemcitabine/Capecitabine

Neoptolemas et al. orchestrated a phase 3, multicenter randomized trial with 730 patients to assess if there is an additional benefit to the addition of capecitabine to gemcitabine. The patients receiving a combination of gemcitabine and capecitabine had a longer median overall survival of 28.0 months, in contrast to 25.5 months in the gemcitabine group. (HR: 0.82; *p* = 0.032) [15]. Although this study achieved its primary endpoint, this failed to show any improvement in relapse-free survival.

#### 3.3.3. FOLFIRINOX

Although adjuvant chemotherapy, including gemcitabine and gemcitabine plus capecitabine, demonstrated improved survival, relapse rates continue to be significantly high, between 69 and 75%, two years postoperatively [14,16,17]. Modified FOLFIRINOX (mFOLFIRINOX) was studied as an adjuvant therapy for pancreatic adenocarcinoma. The median overall survival rate for mFOLFIRINOX was noted to be substantially improved at 54.4 months, compared to 35 months. (95% CI 0.48–0.86, *p* = 0.003) in the gemcitabine arm [18]. Patients receiving mFOLFIRINOX were noted to have higher incidence of grade 3 or 4 adverse events at 75.9%, in contrast to 52.9% in patients who received gemcitabine. Fatigue, diarrhea and sensory neuropathy accounted for the most commonly reported adverse events. However, notably, the rate of grade 3 or 4 diarrhea was much higher in patients treated with FOLFIRINOX compared to patients treated with mFOLFIRINOX. For patients with good functional status following surgical resection, the current recommendation is to add mFOLFIRINOX as an adjuvant treatment. Adjuvant gemcitabine single agent or adjuvant gemcitabine plus capecitabine can be considered for patients who may not be suitable for mFOLFIRINOX.

#### 3.3.4. Gemcitabine/Nab-Paclitaxel

The patients who underwent initial macroscopic tumor resection in the absence of any neoadjuvant treatment were randomized to receive gemcitabine/nab-paclitaxel or gemcitabine monotherapy post-operatively. The study failed to meet its primary endpoint of independently assessing disease-free survival. The median DFS, as evaluated by an independent reviewer, was noted to be 19.4 months for patients in the gemcitabine/nab-paclitaxel group, whereas the median DFS was 18.8 months for the patients with gemcitabine monotherapy arm (HR: 0.88; 95% CI 0.729–1.063, *p* = 0.18) and, hence, the primary endpoint was not achieved [16,19]. However, gemcitabine/nab-paclitaxel demonstrated significantly prolonged investigator-assessed DFS (median DFS 16.6 versus 13.7 months, *p* = 0.02), and significantly prolonged OS (median OS: 41.8 versus 37.7 months, *p* = 0.009). The discrepancy between centrally assessed and investigator-assessed DFS is likely from the challenges in assessing disease recurrence independently without additional pertinent clinical information. In particular, the evaluation of local recurrence and peritoneal lesions by CT or MRI imaging alone after surgical resection is very challenging. This combination can be considered for patients who cannot receive mFOLFIRINOX or gemcitabine/capecitabine.

#### 3.3.5. Adjuvant Chemotherapy following Neoadjuvant Chemotherapy

Patients undergoing pancreatectomy for pancreatic cancer after a minimum of two cycles of neoadjuvant FOLFIRINOX were evaluated in a multicenter, retrospective cohort study by Roessel et al. [20]. A total of 536 patients received neoadjuvant FOLFIRINOX followed by subsequent surgery. Out of these, 343 patients received adjuvant chemotherapy with FOLFIRINOX or a gemcitabine-containing regimen, and 193 patients did not receive any adjuvant therapy. The study indicated that there was no greater advantage in terms of survival for patients who underwent adjuvant chemotherapy as opposed to those who did not (median OS: 29 versus 29 months, *p* = 0.93). Notably, during the multivariate analysis, patients who had positive lymph nodes exhibited improved survival outcomes when administered adjuvant chemotherapy (median OS: 26 versus 13 months; 95% CI 0.22–0.75, *p* = 0.004).

In contrast, another retrospective study showed the survival benefit of adjuvant chemotherapy following multiagent neoadjuvant chemotherapy and resection compared with no adjuvant chemotherapy (26.6 versus 21.2 months, *p* = 0.002) [21]. The survival benefit of adjuvant chemotherapy was observed in patients with any pathological N category and marginal status, in patients younger than 75 years old, in those with a pathological T3 and T4 and in those with moderately or poorly differentiated tumors.

Since these are retrospective studies, further prospective studies are needed to verify the role of adjuvant treatment following neoadjuvant treatment and surgery.

The major adjuvant phase II/III clinical trials are summarized in Table 2.

### 3.4. Metastatic Disease

#### 3.4.1. FOLFIRINOX

The PRODIGE 4 Intergroup randomized trial was a major study defining the treatment paradigm for patients with metastatic PDAC [22]. Patients with metastatic disease who received FOLFIRINOX or gemcitabine monotherapy as upfront treatment. Treatment with FOLFIRINOX showed better overall survival compared to gemcitabine (median OS: 11.1 versus 6.8 months, *p* < 0.001). A subsequent quality of life (QoL) analysis of the study demonstrated that patients receiving FOLFIRINOX had significantly reduced QoL in comparison to gemcitabine. The highest number of severe adverse events were noted in the group that received FOLFIRINOX febrile neutropenia (5.4%), thrombocytopenia (9.1%), diarrhea (12.7%) and sensory neuropathy (9%) while, in the group that received gemcitabine, the incidences were noted to be 1.2%, 3.6%, 1.8% and 0%, respectively [23]. In order to alleviate the side effects from the therapy, the treatment is usually modified by eliminating the 5-FU bolus and a concurrent dose reduction for irinotecan, oxaliplatin or 5-FU infusion. Hence, compared to the conventional dose, modified FOLFIRINOX had a more favorable toxicity profile with comparable efficacy to the standard dose. The original PRODIGE study enrolled patients up to age 75 years, however, the subsequent modified FOLFIRINOX study patients had a relatively broader age range with the oldest patient having an age of 81 years [24,25]. In a subsequent systemic review and meta-analysis of subsequent phase II trials and of series studies evaluating the FOLFIRINOX regimen in metastatic pancreatic cancer, similar results to the initial randomized Phase III trial were noted [26].

#### 3.4.2. Gemcitabine plus Nab-Paclitaxel

The MPACT study included patients with advanced pancreatic cancer who were randomized into two groups; nab-paclitaxel plus gemcitabine or gemcitabine alone. This demonstrated that the combination of nab-paclitaxel and gemcitabine resulted in a statistically significant longer OS (median OS: 8.7 versus 6.6 months, HR = 0.72; 95% CI = 0.62 to 0.83, *p* < 0.001). In the arm of patients that received nab-paclitaxel and gemcitabine, neutropenia was the most commonly observed grade 3 or 4 adverse event with a rate of 38%, while the arm of patients that received gemcitabine monotherapy had a rate of 27%. Amongst the grade 3 or higher non-hematologic adverse events noted with nab-paclitaxel treatment, the most commonly observed ones included fatigue (17%) and peripheral neuropathy (17%) [27,28]. This trial had patients with a wider clinical criteria up including patients aged up to 88 years and a performance status of ECOG 0-2. However, it must be noted that no subgroup analysis evaluating efficacy or toxicity was performed for patients over 75 years, and the patients with ECOG 2 comprised a minority of the trial population in the study. A subsequent phase I/II trial in patients with ECOG 2 showed favorable efficacy and an acceptable safety profile in patients with advanced pancreatic ductal adenocarcinoma [29].

#### 3.4.3. NALIRIFOX

Patients with metastatic PDAC with no prior treatment history were assessed in the NAPOLI-3 trial. In this study, patients were randomized to receive liposomal irinotecan 50 mg/m^2^, 5-FU 2400 mg/m^2^, LV 400 mg/m^2^ and oxaliplatin 60 mg/m^2^ (NALIRIFOX) on days 1 and 15 of a 28-day cycle, or nab-paclitaxel in addition to gemcitabine on days 1, 8 and 15 of a 28-day cycle [30]. When compared to nab-paclitaxel plus gemcitabine, the NALIFIROX group showed a significantly longer overall survival (median OS: 11.1 versus 9.2 months, HR: 0.84; 95% CI 0.71–0.99, *p* = 0.04). Grade 3 or 4 adverse events such as diarrhea (20.3% vs. 4.5%), hypokalemia (15.1% vs. 4%), neutropenia (14.1% vs. 24.5%), nausea (11.9% vs. 2.6%) and anemia (10.5% vs. 17.4%) were more common in the NALIRIFOX group in comparison to the control group.

#### 3.4.4. Gemcitabine plus Albumin-Bound Paclitaxel plus Cisplatin

DNA damage repair deficiencies are an integral part of the pathophysiology in pancreatic cancers, hence, the incorporation of platinum-based chemotherapy has been explored alongside gemcitabine and nab-paclitaxel [31]. A phase I/II clinical trial comprising 25 patients explored the clinical utility of adding cisplatin to the nab-paclitaxel/gemcitabine chemotherapy regimen. The trial demonstrated that 71% of the 24 patients evaluated reached an objective response, including 2 patients who achieved complete response. The median OS and median PFS were 16.4 and 10.1 months, respectively. Hematological adverse events comprised the majority of the grade 3 or 4 treatment adverse events with thrombocytopenia noted in 68% of patients, anemia in 32% and neutropenia in 24%. Despite the encouraging results with a promising overall response rate and progression-free survival, the fact that this was non-randomized and had a small sample size are shortcomings and should be evaluated in further studies to confirm these findings.

#### 3.4.5. Sequential Therapy

A randomized, phase II trial in patients with advanced pancreatic cancer randomized patients to receive either initial treatment with nab-paclitaxel plus gemcitabine on days 1, 8, and 15, followed by modified FOLFOX-6 on day 29 of a 6-week cycle, or with nab-paclitaxel plus gemcitabine on days 1, 8 and 15 of a 4-week cycle [32]. Patients receiving treatment with sequential nab-paclitaxel/gemcitabine followed by FOLFOX showed a significantly better 24-month OS rate of 22.4% compared to 7.6% in the control group (*p* = 0.012). Moreover, the median OS was prolonged in the sequential treatment group at 13.2 months versus 9.7 months in the control group (HR = 0.676; 95% CI 0.483–0.947, *p* = 0.023). Patients receiving a sequential treatment exhibited a greater number of grade ≥ 3 neutropenia (46.1% vs. 24.1%, *p* = 0.004) and grade ≥ 3 thrombocytopenia (23.7% vs. 7.6%, *p* = 0.007) in comparison to the control group.

#### 3.4.6. Maintenance Therapy

The BRCA genes are involved in encoding proteins that are responsible for the repairing of double-stranded DNA breaks through homologous recombination. Almost 4 to 7% of patients diagnosed with pancreatic cancer have inherited mutations including BRCA [33,34,35,36]. A loss of function mutations in the BRCA1 and BRCA2 genes is associated with a higher likelihood of the development of breast and ovarian cancers [37]. Poly (adenosine diphosphate-ribose) polymerase (PARP) inhibitors are drugs which are specifically directed against cells with deficiencies in homologous recombination repair. When cells are deficient in the capability to repair DNA through homologous recombination, PARP enzymes become trapped on DNA at the site of single-strand breaks, which impedes rectification and causes double-strand breaks during cell replication. PARP inhibitors can result in cumulative DNA damage and the ultimate death of tumor cells.

Patients with metastatic pancreatic cancer with a germline mutation in BRCA1 or BRCA2, who had not progressed after receiving platinum-based chemotherapy for at least 16 weeks, were studied in a phase 3 clinical trial. The patients were randomized to receive either maintenance Olaparib at a dose of 300 mg twice daily or a placebo [38]. The Olaparib arm had a significantly prolonged median progression-free survival of 7.4 months in contrast to the placebo arm with 3.8 months (with a HR of 0.53, 95% CI 0.35–0.82, and a *p*-value of 0.004). However, the median OS was similar in the two arms; 18.9 months versus 18.1 months and a *p*-value of 0.68), respectively. Approximately 40% of the patients who received olaparib developed grade ≥ 3 adverse events, with anemia being the most frequent (11%) [39]. It must be considered that the overall survival in this trial may be confounded due to cross-over between the arms and subsequent DNA-damaging chemotherapy.

#### 3.4.7. Subsequent Lines of Therapy

For patients with metastatic pancreatic cancer who have progressed following gemcitabine-based therapy, the first approved treatment is a combination of liposomal irinotecan, 5-fluorouracil and leucovorin. The recommendation is based on results from the NAPOLI-1 trial, which included 417 patients who had progressed after gemcitabine-based therapy. These were randomized into three groups: liposomal irinotecan monotherapy, 5-fluorouracil/leucovorin and liposomal irinotecan/5-fluorouracil/leucovorin [40]. The arm that received liposomal irinotecan/5-fluorouracil/leucovorin had a significantly longer overall survival (median OS: 6.1 months versus 4.2 months) in contrast to the 5-fluorouracil/leucovorin group (HR: 0.67; 95% CI 0.49–0.92, *p* = 0.012). However, higher grade 3 or 4 toxicities, including neutropenia (27% versus 1%), fatigue (14% versus 4%) and diarrhea (13% versus 4%), were noted with liposomal irinotecan/5-fluorouracil/leucovorin treatment compared to 5-fluorouracil/leucovorin.

#### 3.4.8. BRCA1/2 or PALB2 Mutation

The synergistic effect of the PARP inhibitor and of platinum-based chemotherapy was assessed in a randomized phase 2 study including treatment-naive patients with pancreatic cancer with germline BRCA1/2 or PALB2 mutations. These patients received cisplatin plus gemcitabine with or without veliparib. The study demonstrated no significant difference in the objective response rate between the cisplatin/gemcitabine plus veliparib arm (74.1%) and the cisplatin/gemcitabine alone arm (65.2%) with a *p*-value of 0.55. Due to severe hematologic toxicities, 74% of patients with cisplatin/gemcitabine plus veliparib had a dose reduction or drug discontinuation in comparison to 26% with chemotherapy alone. This findings suggest that gemcitabine plus cisplatin may be effective for BRCA1/2 or PALB2 mutated pancreatic cancer, and the addition of veliparib to gemcitabine/cisplatin chemotherapy may be too toxic [41,42].

PARP inhibitors including rucaparib, niraparib and talazoparib are approved for use in various malignancies carrying BRCA mutation such as ovarian, prostrate, breast and pancreatic cancers. Rucaparib was evaluated in a phase II trial as a maintenance regimen for advanced pancreatic cancer (including both metastatic and locally advanced cases) in patients harboring BRCA1, BRCA2 or PALB2 germline or somatic mutations. The trial had a total of 42 patients who had previously received at least 16 weeks of platinum-based chemotherapy without any evidence of disease progression. The results showed that, with maintenance Rucaparib, the median progression-free survival was 13.1 months, and the overall response rate was 41.7%, with 3 patients with complete responses and 12 with partial responses. However, it should be noted that this was not a randomized controlled trial, and therefore subsequent studies are required to fully validate the findings [43].

#### 3.4.9. KRAS Mutations

KRAS is the most common somatic mutation identified in pancreatic ductal adenocarcinoma and is seen in 90% of cases. The commonly observed driver mutations in pancreatic cancer include KRAS, TP53 and SMAD4 [44]. The KRAS protein behaves as a molecular switch modulated by upstream EGFR activation and impacts downstream MAPK and PI3K/mTOR pathways [45]. However, the primary challenge has been the inability to have targetable drugs for the KRAS mutation, attributable mainly to its complicated biochemical characteristics, greater affinity for guanosine triphosphate and smaller number of binding sites. Initially, the inhibition of downstream KRAS mutations was evaluated with MEK inhibitors, but this led to an increased activation of the PI3kinase and failed to show any anticancer activity [46].

KRAS G12C accounts for approximately 1–2% mutations in pancreatic adenocarcinomas. These mutations consist of a unique pocket below the switch II region that leads to selective and irreversible binding, and the subsequent inhibition of oncogenic signaling, by locking it in the inactive GDP-bound state. In a combined phase I/II study, 38 patients, who had previously been administered treatment for metastatic KRAS G12C mutated pancreatic cancer, were given sotorasib 960mg once daily. The study showed that eight patients had a partial response and 21.1% had an objective response rate (95% CI: 9.55–37.32%) [47]. A total of six patients experienced treatment-associated adverse events of grade 3 or greater, including fatigue (two patients), diarrhea (two patients), transaminase elevation (two patients), pleural effusion and pulmonary embolism (one patient each). Nonetheless, sotorasib has clearly shown a clinically significant response in patients with refractory/relapsed KRAS G12C mutated metastatic pancreatic cancer.

KRAS G12D is the most common KRAS mutation in pancreatic adenocarcinoma (45–50%) [48]. Currently, several KRAS-G12D-targeting trials are ongoing. One of these multicenter trials is exploring a novel, proteolysis-targeting chimeric-degrading molecule that is specifically targeting KRAS G12D mutated protein for degradation [49] (NCT05382559). Another phase 1/2 multicenter study is evaluating MRTX1133 in solid malignancies harboring the KRAS G12D mutation. (NCT05737706).

#### 3.4.10. Neurotrophic Tyrosine Receptor Kinase (NTRK) and NRG-1 Gene Fusion

The molecular profiling of tumors has led to the exploration of targetable therapies including rare oncogenic rearrangements which can be drivers of malignancy [50]. NTRK gene fusions have been noted in almost 1% of solid tumors and NTRK inhibitors such as larotrectinib and entrectinib have been shown to have anti-tumor activity, irrespective of tumor type, including pancreatic cancer [51,52].

NRG-1 fusions are noted in <1% cases of pancreatic cancer and clinical studies have shown efficacy with a drug called zenocutuzumab [53].

The major phase II/III clinical trials for metastatic disease are summarized in Table 3.

#### 3.4.11. Immunotherapy

The promising clinical benefit of immune checkpoint inhibitors in various types of solid tumors, such as melanoma and lung cancer, led to the investigation of immunotherapy for pancreatic cancer. The therapeutic effects of durvalumab with or without tremelimumab in patients with metastatic pancreatic ductal adenocarcinoma were explored in a phase 2 trial. The study demonstrated that the blockade of PD-1 alone, or in combination with CTLA-4, did not result in any improvement in efficacy in pancreatic cancer [54].

However, immune checkpoint inhibitors may be efficacious in a subset of patients, while pancreatic cancers with deficient mismatch repair system (dMMR) or high microsatellite instability (MSI-H) are rare, accounting for just 1% of cases [55,56]. In this subset of cancers, immune checkpoint inhibitors such as PD-1/PD-L1-directed therapies have been investigated [55,57]. The KEYNOTE-158 study, a phase II trial, included 22 patients with pancreatic cancer. The patients with previously treated metastatic non-colorectal malignancy received Pembrolizumab intravenously every 3 weeks at a dose of 200 mg. Four patients achieved an objective response (18.2%), including one complete response, and the median duration of response was noted to be 13.4 months [58].

Pancreatic cancer patients who are microsatellite stable but have a high tumor mutational burden (TMB ≥ 10 mut/Mb) have been noted to have a longer survival and stronger anti-tumor immunity, indicating an advantage with use of checkpoint inhibitors [59,60].

A phase 1/2 study was conducted by Reiss et al. to investigate the effects of immune checkpoint inhibitors and the PARP inhibitor combination treatment in platinum-sensitive pancreatic cancer. The study recruited patients with advanced pancreatic cancer whose cancer did not progress after 16 weeks of platinum-based therapy and they received niraparib in combination with either nivolumab or ipilimumab [61]. The arm which received maintenance niraparib plus ipilimumab showed a 6-month progression-free survival rate of 59.6%, while the patients receiving niraparib plus nivolumab showed a rate of 20.6%. It is unclear why ipilimumab demonstrated better anticancer activity than nivolumab in this study. Currently, further studies are underway for the verification of these findings and a better understanding of the superiority of niraparib plus ipilimumab.

IL-10 is considered as one of the major immune suppressive molecules to promote tumor cell proliferation and metastasis. However, recent preclinical studies demonstrated a pegylated IL-10-induced regression of cancer via CD8 T cell activation [62].

The SEQUOIA study aimed to assess whether adding pegilodecakin (a type of interleukin-10 that has been modified to stay in the body longer) to FOLFOX (a chemotherapy regimen) as a second-line treatment would be effective in patients with pancreatic adenocarcinoma who did not respond to gemcitabine [63]. It did not demonstrate any enhancement in objective response rate or overall survival when pegilodecakin was added to FOLFOX as a second-line therapy for gemcitabine-resistant pancreatic adenocarcinoma.

Since chimeric antigen receptor T cell (CAR T) therapy has remarkably improved the clinical outcome of lymphoma, cellular immunotherapy, including tumor infiltrating lymphocyte (TIL) therapy, CAR T and T cell receptor (TCR) T cell therapy, has been studied in pancreatic cancer. Early clinical studies of CAR T targeting CD133, HER2 or mesothelin showed limited anticancer activity [64]. Recently, genetically engineered HLA-restricted TCR T cell therapy targeting KRAS G12D mutations demonstrated a durable response in a patient with metastatic pancreatic cancer [65]. Although further studies are needed to determine the anticancer activity of this approach, this case suggests the major driver mutations of KRAS in pancreatic cancer can be targeted with T cell therapy.

#### 3.4.12. Other Therapies

The tumor microenvironment of pancreatic cancer is rich in hyaluronan, a major component of the extracellular matrix. Pegvorhyluronidase alfa can break down hyaluronan, modulate the tumor microenvironment and enhance the delivery of systemic therapies to the tumor, improving efficacy and leading to independent tumor toxic effects. In order to evaluate the antitumor effects activity of pegvorhyaluronidase alfa in pancreatic cancer with elevated levels of hyaluronan, a phase III study was carried out with pegvorhyaluronidase alfa in combination with gemcitabine/nab-paclitaxel [66]. In the study, patients with hyaluronan-high pancreatic cancer (defined as having ≥50% hyaluronan staining in cancer tissue samples) were randomly assigned to receive either pegvorhyaluronidase alfa or placebo as an adjunct to nab-paclitaxel/gemcitabine,. However, the former did not show any improvement in clinical outcome. Prior to this trial, preclinical data had demonstrated that angiotensin I receptor inhibitors, which block the renin-angiotensin system, could potentially impede tumor growth and metastasis and ameliorate the effectiveness of systemic therapy by increasing drug delivery to the tumor tissue [67]. A phase 2 trial aimed to explore the efficacy of losartan addition (an angiotensin I receptor inhibitor) to eight cycles of FOLFIRINOX followed by chemoradiation, and enrolled 49 patients with locally advanced pancreatic cancer. The primary goal was to assess for improvement in the margin-negative (R0) resection rate for locally advanced pancreatic cancer. Out of the 49 patients, 34 (69%) underwent surgical resection, and 30 of the 49 patients (61%) achieved R0 resection [68]. However, further research is required to confirm these results.

The inhibition of mitochondrial energy metabolism in tumor cells by CPI-613 can lead to the apoptosis, necrosis and autophagy of cancer cells [69]. A randomized phase 3 study aimed to explore the effect of combining CPI-613 with FOLFIRINOX in metastatic pancreatic cancer. The study demonstrated that CPI-613 did not result in any improvement in efficacy [70].

Complementary therapies, such as acupuncture, biofeedback, dietary supplements, massage therapy and meditation, have been conducted in combination with standard systemic treatment to minimize therapy-related toxicities and improve quality of life in cancer patients. Since patients with pancreatic cancer are usually associated with a high symptom burden including cancer pain, cachexia and pancreatic exocrine insufficiency, in addition to cancer therapy-related toxicities, effective supportive care with complementary therapy may improve not only adherence to cancer therapy but also quality of life. Although further prospective studies are needed to investigate the benefit of complementary therapy, early studies suggest complementary therapy including acupuncture, massage therapy, physical activity and nutrition support can reduce symptom burden in pancreatic cancer [71].

## 4. Discussion and Conclusions

Systemic therapy in advanced pancreatic cancer continues to remain a challenging treatment paradigm. Although the outcomes have significantly evolved in the last couple of decades, with combination chemotherapeutic regimens, current standard systemic treatment shows modest anticancer activity and prognosis still remains poor. To improve the clinical outcome of pancreatic cancer, a better understanding of the molecular biology of pancreatic cancer, and the limitations of the current available systemic treatment approaches, is essential. Multidisciplinary approaches, comprehensive germline and somatic gene testing and complementary/supportive care should be considered to improve the clinical outcome and quality of life in patients with pancreatic cancer. Prospectively, understanding the choice of systemic treatment regimen, establishing the best therapeutic sequence, targeted treatment options and personalized medicine, appears to be an upcoming frontier in the field of pancreatic cancer.

## Figures and Tables

**Table 1:** Phase II/III neoadjuvant clinical trials for resectable, borderline resectable and locally advanced disease. BRPC: borderline resectable pancreatic cancer, cape: capecitabine, chemo: chemotherapy, CI: confidence interval, CRT: chemoradiation, erlo: erlotinib, FFN: FOLFIRINOX, gem: gemcitabine, LAPC: locally advanced pancreatic cancer, mo: month, neoadj: neoadjuvant, nab-p: nab-paclitaxel, OS: overall survival and RPC: resectable pancreatic cancer.

Treatment Regimen	Phase	Population	*N*	Primary End Point	Ref.
FFN	II	BRPC	48	R0 resection rate: 65%	[4]
immediate surgery vs. neoadj treatment (gem/cape, FFN or CRT)	II	BRPC	90	Recruitment rate: 2.16 patients/month Resection rate: surgery-68% (R0:14%) vs. neoadj-55% (R0: 23%), *p* = 0.33 (resection), *p* = 0.49 (R0)	[6]
FFN vs. gem/nab-p	II	RPC	147	2-year OS: FFN-47% vs. gem/nab-p-48%	[7]
First randomization Gem vs. Gem/Erlo Second randomization Chemo vs. CRT	III	LAPC	442	OS (median): gem-13.6 mo vs. gem/erlo 11.9 mo, *p* = 0.09; chemo-16.5 mo vs. CRT-15.2 mo, *p* = 0.83	[8]
Gem/Nab-p	II	LAPC	107	Time to treatment failure: 9 mo (90% CI 7.3–10.1)	[9]
FFN vs. gem/nab-p	II	LAPC	126	1-year OS: 77.4% vs. 82.5% (HR, 1.1; 95% CI: 0.73–1.65)	[10]

**Table 2:** Phase II/III adjuvant clinical trials in patients with surgically resected pancreatic cancer. 5FU: fluorouracil, CI: confidence interval, CRT: chemoradiation, DFS: disease-free survival, FFN: FOLFIRINOX, gem: gemcitabine, HR: hazard ratio, mo: month, nab-p: nab-paclitaxel and OS: overall survival.

Treatment Regimen	Phase	*N*	Primary End Point	Reference
Gem vs. observation alone	III	368	DFS (median): gem-13.4 mo vs. 6.7 mo (HR, 0.55; 95% CI 0.44–0.69, *p* < 0.001)	[14]
Gem vs. 5FU (all patients received CRT with 5FU)	III	451	OS (median): Gem-20.5 mo vs. 5FU-16.9 mo (HR, 0.82; 95% CI 0.65–1.03, *p* = 0.09)	[15]
FFN vs. gem	III	493	DFS (median): FFN-21.6 mo vs. gem-12.8 mo (HR, 0.58; 95% CI 0.46–0.73, *p* < 0.001)	[18]
5FU vs. Gem	III	1088	OS (median): 5FU-23.0 mo vs. gem-23.6 mo (HR, 0.94; 95% CI 0.81–1.08, *p* = 0.39)	[16]
Gem/nab-p vs. gem	III	866	Independently assessed DFS (median): gem/nab-p-19.4 mo vs. gem-18.8 mo (HR, 0.88; 95% CI 0.73–1.06, *p* = 0.18)	[19]

**Table 3:** Phase II/III clinical trials in patients with advanced/metastatic pancreatic cancer. 5FU: fluorouracil, CI: confidence interval, CR: complete response, FFN: FOLFIRINOX, gem: gemcitabine, HR: hazard ratio, lipo iri: liposomal irinotecan, mo: month, nab-p: nab-paclitaxel, OR: objective response, OS: overall survival, PFS: progression-free survival and RR: response rate.

Treatment Regimen	Phase	Population	*N*	Primary End Point	Reference
FFN vs. Gem	III	Treatment naïve metastatic disease	342	OS (median): FFN-11.1 mo vs. gem-6.8 mo (HR, 0.57; 95% CI 0.45–0.73, *p* < 0.001)	[23]
Gem/nab-p vs. gem	III	Treatment naïve metastatic disease	861	OS (median): gem/nab-p-8.5 mo vs. gem-6.7 mo (HR, 0.72; 95% CI 0.62 to 0.83, *p* < 0.001)	[27]
NALIRIFOX vs. Gem/Nab-p	III	Treatment naïve metastatic disease	770	OS (median): NALIRIFOX-11.1 mo vs. gem/nab-p-9.2 mo (HR, 0.84; 95% CI 0.71–0.99, *p* = 0.04)	[30]
Gemcitabine/nab-paclitaxel/cisplatin	IB/II	Treatment naïve metastatic disease	25	CR rate: 8%	[31]
Gem/Nab-P followed by FOLFOX (sequential) vs. gem/nab-p	II	Treatment naïve metastatic disease	157	12 mo OS: sequential-55.3% vs. gem/nab-p-35.4% (*p* = 0.016)	[32]
Maintenance olaparib vs. placebo	III	BRCA mutated disease	154	PFS (median): olaprib-7.4 mo vs. placebo-3.8 mo (HR, 0.53; 95% CI 0.35–0.82, *p* = 0.004)	[38,39]
Lipo iri/5FU vs. 5FU	III	Refractory metastatic disease	417	OS (median): lipo iri/5FU-6.1 mo vs. 5 FU-4.2 mo (HR, 0.67; 95% CI 0.49–0.92, *p* = 0.012)	[40]
Gem/cisplatin/veliparib vs. gem/cisplatin	II	Treatment naïve *BRCA* or *PALB2* mutated disease	50	RR: gem/cisplatin/veliparib-74.1% vs. gem/cisplatin-65.2% (*p* = 0.55)	[42]
Maintenance rucaparib	II	*BRCA* or *PALB2* mutated disease	42	6-month PFS rate: 59.5%	[43]
Sotorasib	II	KRAS G12C mutated refractory disease	38	centrally confirmed OR rate: 21%	[47]
